# Chemical Vapor Deposition-Fabricated Manganese-Doped and Potassium-Doped Hexagonal Tungsten Trioxide Nanowires with Enhanced Gas Sensing and Photocatalytic Properties

**DOI:** 10.3390/nano12071208

**Published:** 2022-04-04

**Authors:** Pin-Ru Chen, Hsuan-Wei Fu, Shu-Meng Yang, Kuo-Chang Lu

**Affiliations:** 1 Department of Materials Science and Engineering, National Cheng Kung University, Tainan 701, Taiwan; kitty110384@gmail.com (P.-R.C.); n56091556@gs.ncku.edu.tw (H.-W.F.); n56074287@gs.ncku.edu.tw (S.-M.Y.); 2 Core Facility Center, National Cheng Kung University, Tainan 701, Taiwan

**Keywords:** tungsten oxide, nanowire, chemical vapor deposition, doping, hexagonal, photocatalysis, gas sensor

## Abstract

Owing to its unique and variable lattice structure and stoichiometric ratio, tungsten oxide is suitable for material modification; for example, doping is expected to improve its catalytic properties. However, most of the doping experiments are conducted by hydrothermal or multi-step synthesis, which is not only time-consuming but also prone to solvent contamination, having little room for mass production. Here, without a catalyst, we report the formation of high-crystallinity manganese-doped and potassium-doped tungsten oxide nanowires through chemical vapor deposition (CVD) with interesting characterization, photocatalytic, and gas sensing properties. The structure and composition of the nanowires were characterized by transmission electron microscopy (TEM) and energy-dispersive spectroscopy (EDS), respectively, while the morphology and chemical valence were characterized by scanning electron microscopy (SEM) and X-ray photoelectron spectroscopy (XPS), respectively. Electrical measurements showed that the single nanowires doped with manganese and potassium had resistivities of 1.81 × 0^−5^ Ω·m and 1.93 × 10^−5^ Ω·m, respectively. The doping contributed to the phase transition from monoclinic to metastable hexagonal for the tungsten oxide nanowires, the structure of which is known for its hexagonal electron channels. The hexagonal structure provided efficient charge transfer and enhanced the catalytic efficiency of the tungsten oxide nanowires, resulting in a catalytic efficiency of 98.5% for the manganese-doped tungsten oxide nanowires and 97.73% for the potassium-doped tungsten oxide nanowires after four hours of degradation of methylene blue. Additionally, the gas sensing response for 20 ppm of ethanol showed a positive dependence of doping with the manganese-doped and potassium-doped responses being 14.4% and 29.7%, respectively, higher than the pure response at 250 °C. The manganese-doped and potassium-doped tungsten oxide nanowires are attractive candidates in gas sensing, photocatalytic, and energy storage applications, including water splitting, photochromism, and rechargeable batteries.

## 1. Introduction

With the development and advancement of industry, people’s lives are becoming more diverse and convenient; however, this also brings global warming and environmental pollution, making green energy and sustainable development an important issue that this generation must face and find feasible solutions in technology. Photocatalysis is an effective approach to solve environmental pollution by absorbing solar energy through semiconductor materials, stimulating electrons inside the material to the conductive band, and leaving holes in the valence band, which can decompose toxic organic pollutants by the separated electron–hole pairs to conduct oxidation and reduction reactions. In addition, gas sensing devices can be used to monitor the air quality through adsorption and desorption of the target gas and metal oxide semiconductors [1]; as the depletion layer of the material increases or decreases, the material resistance would change accordingly, and the surrounding target gas can be detected with high reusability and low energy loss.

Due to their high specific surface area and oxygen-vacancy-rich properties, tungsten oxide nanowires are of great interest for future semiconductor applications, such as electrodes for rechargeable storage applications [2,3,4], electrochromic devices, field emitters [5], photochemical hydrogen production [6], photocatalytic materials for degradation of organic pollutants [7], and gas sensors [8,9]. However, the excessively high electron–hole recombination rate limits the availability, weakening the oxidation reduction ability and the catalytic effect. To enhance the performance of tungsten oxide nanowires for subsequent applications, several strategies are commonly used, including morphology control [10], metal doping [11], precious metal modification [12], and construction of composite materials [9]. In particular, doping is an effective method to adjust the structure of the electron band; the position of the valence and conduction band can be adjusted by metal replacement. Additionally, doping may cause changes in the crystal growth direction, resistivity, and even phase transformation; therefore, adjusting the band gap to increase the absorption efficiency of visible light, or adding impurities to hinder the recombination of electron–hole pairs is expected to be effective. In addition, tungsten oxide has a multifaceted lattice structure system [13], providing much room for material modification; doping with impurity atoms can supply charge traps and act as scattering centers for carriers, making the life cycle of the electron–hole pair longer. Therefore, the study on the correlation between doping and changes in the lattice structure and physical properties of tungsten oxide materials is essential for future applications. 

Tungsten oxide usually appears as tungsten dioxide (WO_2_) and tungsten trioxide (WO_3_). At 1 atm and room temperature, tungsten trioxide is light yellow with the structure of monoclinic; the monomer is a WO_6_ octahedron, where the W^6+^ ion occupies the middle site of the octahedron, while the oxygen ions occupy the other eight sites of the octahedron. The monomers share the oxygen ions and form a ReO_3_ structure, similar to a perovskite structure. Defects or oxygen vacancies lead to the chemical formula of WO_3-x_, which can be used in photocatalytic applications [14,15]. Combining other photocatalytic materials, such as graphene, may contribute to better photodegradation performance [16]. Moreover, through controlling the annealing temperature and atmosphere, the monoclinic structure may be converted to a metastable hexagonal tungsten trioxide (h-WO_3_) structure that can exist at room temperature [17]. The special structure of h-WO_3_ provides crystal tunnels for the intercalation of small cations, which may be applied in specific capacitance [18,19]. 

Most of the metal-doped tungsten oxide nanowires are synthesized by the hydrothermal method [11,20] and solvent heat method [21], while in this work, we used a three-zone tube furnace to grow nanowires by the CVD method [22,23,24], which has fewer steps than the common doping methods. Moreover, the products here are less likely to be contaminated by the reactive solute solvent. Organic pollutant methylene blue was then degraded for four hours using a tera solar light simulator. Afterwards, single manganese-doped and potassium-doped tungsten oxide nanowires were connected to gold electrodes with FIB for gas sensing devices and 20 ppm of ethanol was sensed at different temperatures. We expect that the photocatalytic efficiency and gas sensing response of the modified tungsten oxide nanowires will be significantly improved as compared with the undoped tungsten oxide nanowires.

## 2. Materials and Methods

### 2.1. Synthesis of Tungsten Oxide Nanowires

In this study, single-phase tungsten trioxide nanowires were grown by thermal vapor deposition in a three-zone tube furnace. Tungsten oxide powder was placed in an alumina boat crucible in the first heating zone of the tube furnace, while a silicon substrate was placed at the third heating zone. The first and second heating zones were set at 1140 °C with the third at 1060 °C for 2 h. Argon gas with a flow rate of 100 sccm was used as the carrier gas. The tungsten trioxide nanowires were obtained after 6 h of cooling naturally to room temperature.

### 2.2. Synthesis of Manganese-Doped and Potassium-Doped Tungsten Oxide Nanowires

A single step growth method was used for doping. Manganese chloride (MnCl_2_, Alfa Aesar, Haverhill, MA, USA, purity 97%) was placed in the first heating zone and tungsten trioxide powder was placed in the second heating zone. The first and second heating zones were set at 1140 °C, while the third zone was set at 1060 °C. Then, 10 sccm of oxygen and 90 sccm of argon were introduced as the reaction gas and carrier gas, respectively. The temperature was held for six hours and then cooled down to room temperature naturally. As for synthesis of potassium-doped tungsten oxide nanowires, potassium iodide (KI, Sigma-Aldrich, St. Louis, MO, USA, purity 99.5%) was placed in the first heating zone of the tube furnace tube, while tungsten trioxide powder was placed in the third heating zone with 100 sccm of argon as the carrier gas. The rest of the procedures were the same as the manganese doping experiment. Investigation and characterization of the synthesized WO_3_, Mn-doped, and K-doped WO_3_ nanowires were conducted with AFE-SEM (Zeiss Auriga, CarlZeiss, Jena, Germany) HR-TEM (JOEL JEM-2100F CS STEM, Tokyo, Japan), (EDS (Bruker, Billerica, MA, USA), and XPS (ULVAC-PHI 5000 Versaprobe, Chigasaki, Kanagawa, Japan).

### 2.3. Electrical Measurements

The single nanowire measurement method we utilized here was previously reported [25]. First, we deposited a 300 nm-thick silicon dioxide layer on ultrasonically cleaned silicon substrates as an insulation layer by an e-beam evaporation system (ULVAC VT1-10CE). Then, we pasted a copper mesh as a mask and deposited a 200 nm-thick silver layer by e-beam evaporation as a conduction layer. After that, we obtained the nanowires from specimens by an ultrasonic oscillator and dripped the nanowires to the prepared silver-coated substrate, as shown in Figure S3. The nanowire was then connected to the electrodes by a dual-beam focused ion beam system (FEI Helios G3CX). To avoid the facet effect [26], the platinum pad should be thick enough to cover the nanowire, as shown in Figure S4. Finally, we measured the nanowire resistance by connecting four probes to the four contacts.

### 2.4. Preparation of Gas-Sensing Micro-Devices

Silicon dioxide was deposited as the insulating layer, a steel wire was glued as the photomask, and chromium was deposited as the adhesive layer between the electrode and substrate with gold deposited as the conductive electrode. The prepared tungsten oxide nanowires were put into a glass vial and some DI water was added inside. The nanowires were shaken down with an ultrasonic oscillator and dripped onto the prepared micro-devices. Finally, a double-beam focused ion beam system (FEI Helios G3CX) was used to connect both ends of the nanowires to the electrodes with platinum.

### 2.5. Photodegradation Experiments

Here, 50 ppm of methylene blue was used as the source of contaminants, and photodegradation experiments were conducted on tungsten oxide nanowires, and manganese-doped and potassium-doped tungsten oxide nanowires, respectively, using a xenon lamp simulating sunlight as the light source for full-spectrum irradiation (320–780 nm). Prior to the experiment, the specimen was placed in a dark box to ensure the equilibrium of adsorption and desorption between the specimen and the solution, and then the experiment was conducted for four hours with 30 min intervals.

### 2.6. Gas Sensing Experiments

Here, 300 sccm of a hydrogen-oxygen mixture of ordinary air was continuously passed into the chamber; thus, the inlet-extraction of the chamber reached equilibrium. As the heaters in the chamber were warmed up to the specified operating temperatures, a fixed voltage of 1 V was given to the device to start measuring the change in current. When the current was stabilized, we turned on 20 ppm of ethanol at the flow rate of 300 sccm and turned off the air for the gas sensing measurement.

## 3. Results

### 3.1. Synthesis and Characterization of WO_3_, Mn-Doped WO_3_, and K-Doped WO_3_ Nanowires 

**Figure 1:**
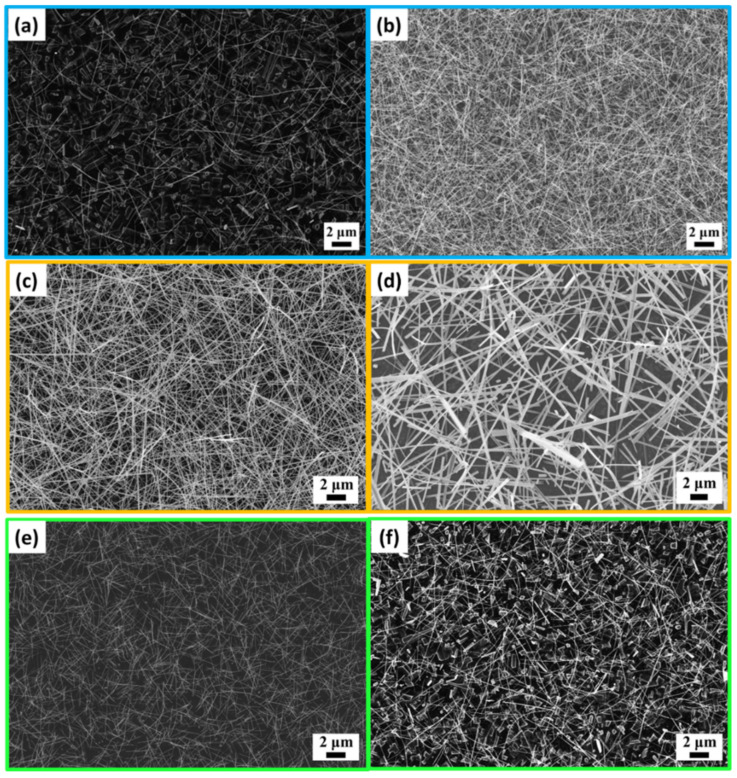
SEM images of Mn-doped and K-doped WO_3_ nanowires. (**a**,**b**) Mn-doped WO_3_ nanowires with different quantities of precursors: (**a**) 0.02 g of MnCl_2_ and (**b**) 0.03 g of MnCl_2_; (**c**,**d**) K-doped WO_3_ nanowires with different quantities of precursors: (**c**) 0.03 g of KI and (**d**) 0.04 g of KI; (**e**,**f**) Mn-doped WO_3_ nanowires with different oxygen partial pressures: (**e**) 0.09 torr and (**f**) 0.18 torr.

Figure 1 shows SEM images of Mn-doped and K-doped WO_3_ nanowires. In this experiment, 0.15 g of WO_3_ powder was used as the precursor base for Mn doping and K doping experiments. From Figure 1a,b, it can be observed that for the nanowires, with 0.02 g of MnCl_2_ powder, the nanowire density was lower, and the nanowire diameter was diverse, while with 0.03 g of MnCl_2_ powder, the nanowire density was higher, and the nanowire diameter was similar. More MnCl_2_ powder led to more gas particles, a larger frequency of collisions per second, and a shorter average free radius of the particles; thus, they would be in the vicinity of the nucleation sites for deposition, increasing the formation of nanowires. On the contrary, less MnCl_2_ powder contributed to a larger average free radius and fewer nanowires. Figure 1c,d show K-doped WO_3_ nanowires with different quantities of precursors. With 0.03 g of KI powder, both the density and aspect ratio of the nanowires were high, while with 0.04 g of KI powder, coarsening appeared due to the high vapor pressure of the precursor.

To solve the low lattice suitability of manganese ions to tungsten oxide, oxygen was introduced to assist in the formation of manganese oxides; thus, manganese ions could be successfully doped in the tungsten oxide lattice. Based on Figure 1e,f, showing Mn-doped WO_3_ nanowires with different oxygen partial pressures, at the oxygen partial pressure of 0.09 torr, fewer bulk films were generated, and the nanowire density was higher, but the nanowires were mostly short and thin; at the oxygen partial pressure of 0.18 torr, although the aspect ratio was higher, many bulk films were generated, and the nanowire density was also lower. This may be attributed to the fact that more oxygen decreased the average free radius of tungsten oxide vapor, resulting in a shorter diffusion distance.

**Figure 2:**
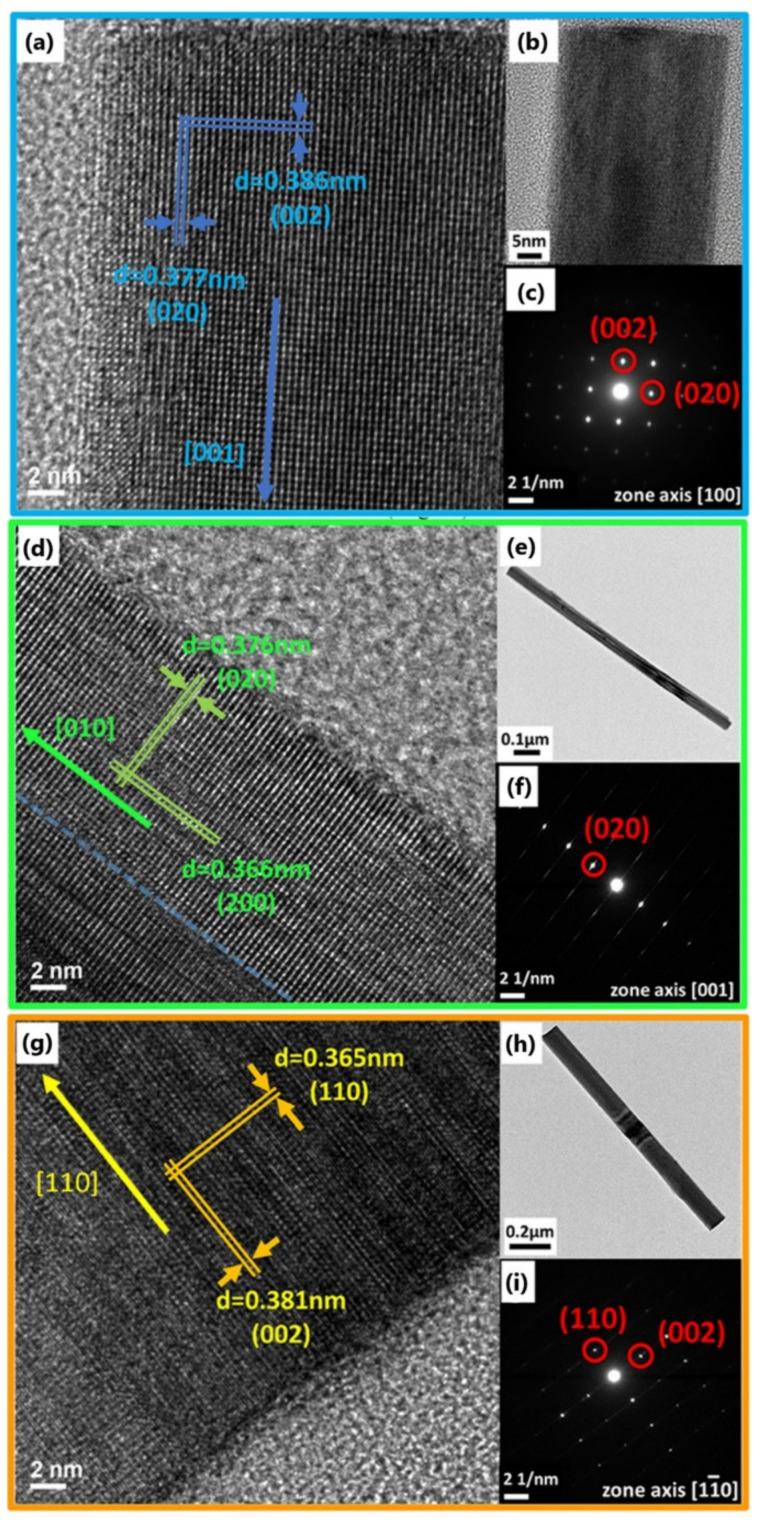
TEM analysis of nanowires. (**a**–**c**) TEM analysis of WO_3_ nanowire: (**a**) HRTEM image, (**b**) low-resolution image, and (**c**) SAED pattern. (**d**–**f**) TEM analysis of Mn-doped WO_3_ nanowire: (**d**) HRTEM image, (**e**) low-resolution image, and (**f**) SAED pattern. (**g**,**h**) TEM analysis of K-doped WO_3_ nanowire: (**g**) HRTEM image, (**h**) low-resolution image, and (**i**) SAED pattern.

The synthesized tungsten oxide nanowires were analyzed by TEM, as shown in Figure 2. In Figure 2a–c, high-resolution transmission microscopy (HRTEM) and selected-area electron diffraction (SAED) analysis showed that the interplanar spacings were 0.386 nm and 0.377 nm, corresponding to (002) and (020); thus, it can be confirmed that the structure was monoclinic, and the growth direction was [001].

TEM analysis of the manganese-doped tungsten oxide nanowires revealed that in addition to the three peaks between 23° and 25° from the original tungsten oxide nanowires, a strong peak was found at 28.11°, which corresponds to the hexagonal tungsten trioxide (No. 085-2459) plane in the JCPDS database. It is concluded that the phase change of the original monoclinic crystal occurred during the doping process [27,28]. In the HRTEM image of the manganese-doped tungsten oxide nanowires, as shown in Figure 2d–f, the interplanar spacings were 0.376 nm and 0.366 nm, corresponding to (020) and (200) crystal planes of the monoclinic phase, respectively; the presence of a clear interface can be observed, contributing to the stacking fault, as indicated by the blue dashed line in Figure 2d, representing the presence of defects in the manganese-doped tungsten oxide nanowires. The SAED showed the reflection from a single crystal plane group as the superposition of different crystal planes in the same direction leads to a weakening of the signal in the other direction. EDS analysis revealed 0.87% of Mn, indicating successful doping of Mn, as shown in Figure S1. Figure S2 shows the EDS mapping of the Mn-doped and K-doped tungsten oxide nanowires.

The HRTEM and SAED images in Figure 2g–i show that the interplanar spacings were 0.365 nm and 0.381 nm, corresponding to (110) and (002) crystal planes and that the growth direction was [110], confirming that the lattice structure was hexagonal. As shown in Figure S1, the ratio of potassium to tungsten (K/W) in EDS was 0.296, coherent with the stoichiometric ratio of metallic elements in JCPDS card No. 081-0005; the chemical formula was written as K_0.333_W_0.944_O_3_.

**Figure 3:**
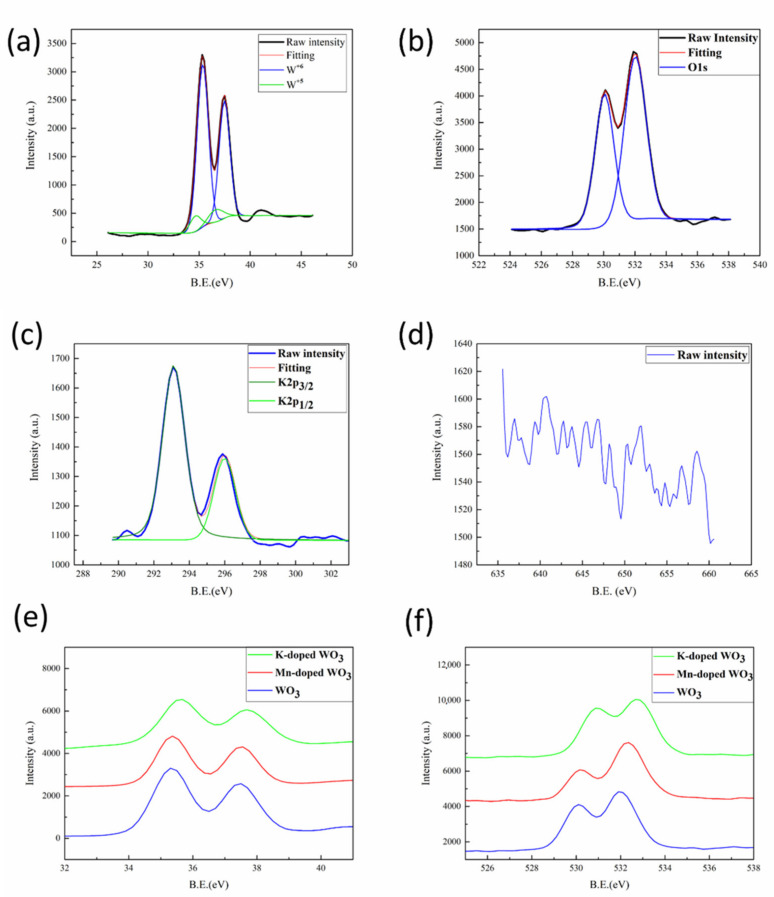
XPS analysis for the undoped tungsten oxide nanowires: (**a**) W4f and (**b**) O1s; XPS analysis for K-doped tungsten oxide nanowires: (**c**) K2p; XPS analysis for Mn-doped tungsten oxide nanowires: (**d**) Mn2p; and XPS comparison of the three kinds of nanowires: (**e**) W4f and (**f**) O1s.

For the chemical valence analysis of the elements in the nanowires, binding energies and relative intensities were obtained by X-ray photoelectron spectroscopy, as shown in Figure 3. For undoped tungsten oxide nanowires, the XPS peaks shown in Figure S8 could be fitted as a mixture of W^6+^ and W^5+^, which is consistent with the binding energy state of oxygen vacancies. In Figure 3a, the binding energies of hexavalent tungsten W4f_7⁄2_ and W4f_5⁄2_ corresponded to 35.4eV and 37.5eV, while the binding energies of pentavalent tungsten W4f_7⁄2_ and W4f_5⁄2_ corresponded to 34.7eV and 36.4eV. Comparing Figure S8a,c, we could find the intensity decrease of W^6+^ and the intensity increase of W^5+^ after doping, demonstrating the increase in oxygen vacancies [15]. The binding energy of O1s corresponded to 530.1 eV; there was a peak at 532.1 eV, resulting from O^2−^, O^−^, and OH^-^ signals based on oxygen vacancies [22], which may be attributed to surface contaminants, such as water molecules attached to the specimen or adsorbed oxygen from the air [29], as shown in Figure 3b. Similarly, the W4f peaks of all metal-doped tungsten oxide nanowires exhibited two major peaks after deconvolution, belonging to W4f_7/2_ and W4f_5/2_ orbitals in the W^6+^ state and two low-intensity peaks in the W^5+^ state. Figure 3c shows the deconvolution for potassium, which corresponded to the K2p_3/2_ orbital at 293.1 eV and the K2p_1/2_ orbital at 296.0 eV. In addition, the peaks of Mn ion-based compounds were not well identified, as shown in Figure 3d, suggesting that Mn ions might have entered the WO_3_ lattice; low doping concentration and few Mn ions on the surface contributed to weak signals, which is similar to previous studies, where the XPS signal peaks were not discernible at doping concentrations less than 1% [30,31]. As XPS is sensitive to the local chemical environment and oxidation state in the surface composition of the material, it was not difficult to find the displacement of the XPS peak according to the chemical changes, as shown in Figure 3e,f; during the doping process, chemical reactions occurred between the material and the doping metals, which changed the chemical state and the relative ion size. The superimposed W4f spectra can be interpreted as a significant shift in the binding energy of the W^6+^ state toward higher binding energies after doping, which indicates that these doped metal ions were successfully doped as impurity atoms replacing W atoms or embedded in the WO_3_ lattice, resulting in a change in the chemical bond strength between the original tungsten atom and oxygen.

In Table 1, the electrical measurements showed that the resistivity of the single nanowires doped with manganese and potassium was measured to be 1.81 × 10^−5^ Ω·m and 1.93 × 10^−5^ Ω·m, respectively, which were much higher than those of the undoped wires (8.27 × 10^−6^ Ω·m), confirming the effect of increasing resistivity due to doping. The resistivity increased after doping as the doping metal ions became a barrier for electron transmission, particularly for potassium-doped tungsten oxide nanowires, which had the highest resistivity. Figures S5–S7 show the I–V measurements of undoped WO_3_ nanowire, Mn-doped WO_3_ nanowire, and K-doped WO_3_ nanowire, respectively.

### 3.2. Photodegradation of Methlene Blue

The methylene blue acted as a pollutant in the experiment, while the doped nanowires and undoped nanowires acted as catalysts. Under the exposure of light, the energy over the band gap of the tungsten oxide nanowire excited the electrons in the valance band (VB) into the conduction band (CB), leaving holes in the valance band. The electrons were reducing agents, while the holes were oxidizing agents. The nanowires reacted with the oxygen and water molecules in the air, generating superoxide radicals and hydroxide radicals, which would degrade methylene blue into water and carbon dioxide. 

**Figure 4:**
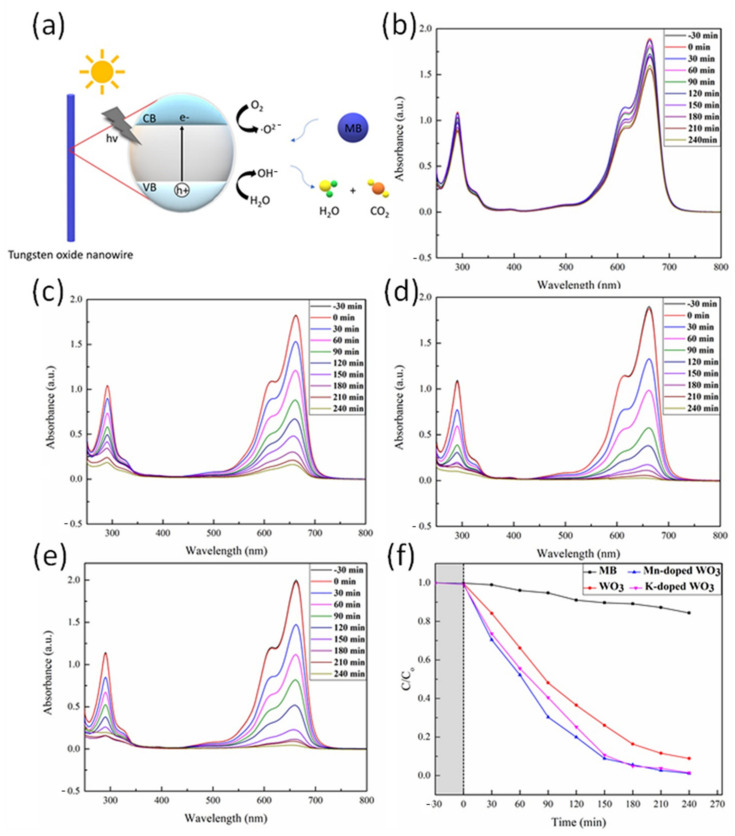
Photodegradation of methylene blue with WO_3_ NW, Mn-doped WO_3_ NW, and K-doped WO_3_ NW. (**a**) Schematic illustration of photodegradation mechanism. (**b**–**e**) UV-Vis results: (**b**) MB, (**c**) undoped WO_3_, (**d**) Mn-doped WO_3_, and (**e**) K-doped WO_3_. (**f**) Line graph of photodegradation of methylene blue.

Figure 4a shows a schematic illustration of the photodegradation mechanism, Figure 4b–e show UV–Vis results of the photodegradation, and Figure 4f shows the line graph of photodegradation studies, where the gray area represents the blank test of methylene blue before illumination on the specimen. From the line graph, the photocatalytic efficiency of the manganese-doped and potassium-doped tungsten oxide nanowires was better than that of the undoped tungsten oxide nanowires. The maximum difference between the manganese-doped and potassium-doped tungsten oxide nanowires was at 150 min of degradation. The manganese-doped tungsten oxide nanowires had a methylene blue concentration difference of 17.5% to the undoped ones, while the potassium-doped tungsten oxide nanowires had almost the same concentration with the undoped nanowires. After 150 min of degradation, both degradation rates began to stabilize with time, in accordance with the trend of methylene blue photodegradation.

The doping improved the efficiency of photocatalysis for two reasons: the effect of doping with impurity atoms and the structural effect due to the phase change caused by doping. The doping brought abundant electrons; thus, more electrons were separated and accumulated in the conduction band of tungsten oxide after illumination; holes were left in the valence band of tungsten oxide; the doping changed the internal energy band structure of the original tungsten oxide material. The impurity atoms became charge traps and electron–hole pair recombination obstruction centers, making the recombination of electron–hole pairs slower, which could be inferred from previous studies [32,33]. Furthermore, doping of manganese or potassium brought a phase change from the monoclinic phase to hexagonal phase, as the enhanced photocatalytic performance resulted from the activation of photogenerated carriers to interact with oxygen and water molecules on the surface of tungsten oxide nanowires. The special electron transfer channels of the hexagonal tungsten oxide phase could improve the interfacial charge transfer and increase the photochemical interaction.

### 3.3. Gas Sensing Mechanism

There are two gas sensing models at temperatures above 500 °C and below 500 °C. The temperature above 500 °C corresponds to the oxygen vacancy mechanism model, where the target gas will interact with the crystal lattice of the metal oxide and form oxygen vacancies. The oxygen vacancies generate electrons to metal oxide, which will increase the concentration of free electrons in the metal oxide and decrease the resistance [34].

**Figure 5:**
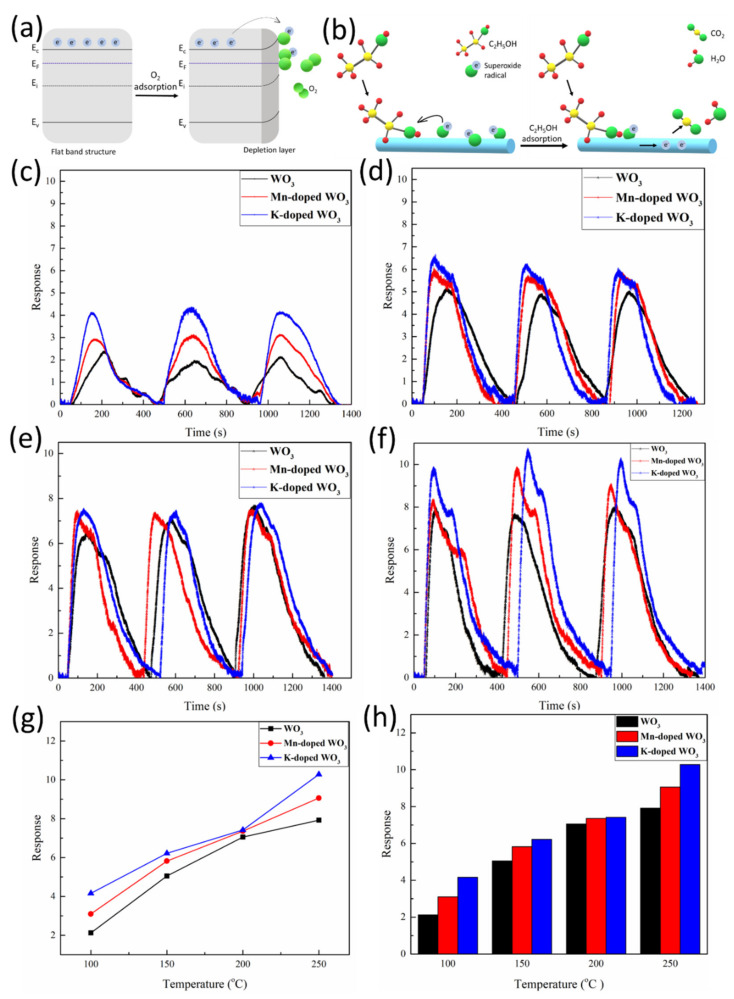
Gas sensing of the nanowires to 20 ppm of ethanol. (**a**,**b**) Schematic illustration of gas sensing mechanism: (**a**) Oxygen adsorbed to the surface of the nanowire to form superoxide radicals. (**b**) Gas sensing mechanism for ethanol. (**c**–**f**) Dynamic gas sensing curves of WO_3_ NW, Mn-doped WO_3_ NW, and K-doped WO_3_ NW at 100 °C, 150 °C, 200 °C, and 250 °C to 20 ppm of ethanol, respectively: (**c**) 100 °C, (**d**) 150 °C, (**e**) 200 °C, and (**f**) 250 °C. (**g**) Line graph of the sensitivity of WO_3_ NW, Mn-doped WO_3_ NW, and K-doped WO_3_ NW to 20 ppm of ethanol at different temperatures. (**h**) Bar graph of the sensitivity of WO_3_ NW, Mn-doped WO_3_ NW, and K-doped WO_3_ NW to 20 ppm of ethanol at different temperatures.

The other model is the oxygen ion adsorption, which is the case here. The oxygen adsorbed on the surface of tungsten oxide nanowires formed different valence oxygen ions, such as O_2_^−^, O^−^, and O^2−^, after trapping free electrons from the conduction band [35,36], as shown in Figure 5a. The oxygen also formed an electron depletion layer on the surface of tungsten oxide nanowires, which narrowed the electron transport channel and increased the overall resistance of the material. The path of oxygen adsorption can be expressed by the following equations:

O_2(gas)_ → O_2(ads)_ (1)

O_2(ads)_ + e^−^ → O_2_^−^ (2)

O_2_^-^ + e^−^ → 2O^-^ (3)

O^−^ + e^−^ → O^2−^ (4)

After interaction with a reducing gas, the electrons were released back to the nanowire, resulting in the resistance decrease of the nanowire; the mechanism is shown in Figure 5b. The reaction path is described by the following equations:

C_2_H_5_OH_(gas)_ → C_2_H_5_OH_(ads)_ (5)

C_2_H_5_OH _(ads)_ + 4O_2_^−^_(ads)_ → 4CO_2(gas)_ + 6H_2_O_(gas)_ + 4e^−^ (6)

C_2_H_5_OH _(ads)_ + 6O^−^_(ads)_ → 2CO_2(gas)_ + 3H_2_O_(gas)_ + 6e^−^ (7)

C_2_H_5_OH _(ads)_ + 3O^2−^_(ads)_ → 2CO_2(gas)_ + 3H_2_O_(gas)_ + 6e^−^ (8)

### 3.4. Gas Sensing Properties

The gas response (S) is presented by:

S = [1-(R_g_/R_a_)] × 100 (for reductive gas) (9)

S = [(R_g_/R_a_)−1] × 100 (for oxidative gas) (10)

where R_g_ and R_a_ are the resistance of the nanowire under target gas and air ambience, respectively. Tungsten oxide is an n-type semiconductor and the main charge transfer carrier is the electron; with exposure to an oxidizing gas atmosphere, including O_2_, NO_2_, and CO_2_, the electrical resistance will increase and the conductivity will decrease; with exposure to a reducing gas atmosphere, such as CO, H_2_, ethanol, and acetone, the electrical resistance will decrease and the conductivity will increase. Figure 5c–h show the gas sensing results of tungsten oxide nanowires, manganese-doped tungsten oxide nanowires, and potassium-doped tungsten oxide nanowires at 100 °C, 150 °C, 200 °C, and 250 °C with 20 ppm of ethanol. It is clear that the sensitivity of the three different tungsten oxide nanowires increased with temperature. Moreover, the gas sensing sensitivity of the doped tungsten oxide nanowires was better than that of the undoped tungsten oxide nanowires; at 250 °C, the response of tungsten oxide nanowires, manganese-doped tungsten oxide nanowires, and potassium-doped tungsten oxide nanowires was 7.92, 9.06, and 10.28, respectively. Potassium-doped tungsten oxide nanowires showed an approximately 30% higher response to 20 ppm of ethanol than undoped tungsten oxide nanowires. This may be attributed to the following factors: (1) Doped metal ions inhibited the lattice growth in tungsten oxide nanowires; with smaller grain size, there would be more grain boundaries, resulting in a shortening of the average free radius of electrons more easily trapped by oxygen in the air to form superoxide radicals. (2) The doping led to a phase transition from monoclinic to hexagonal tungsten oxide nanowires; the hexagonal and trigonal tunneling structures allow faster electron transport.

## 4. Conclusions

In this work, tungsten oxide nanowires with good crystallinity were successfully synthesized in a three-zone tube furnace; manganese-doped and potassium-doped tungsten oxide nanowires with excellent morphology, and high density and aspect ratio were grown by chemical vapor deposition through a single step. Doping of manganese or potassium structurally changed the tungsten oxide nanowire from a stable monoclinic phase to hexagonal phase. XPS analysis showed that the binding energies of tungsten and oxygen atoms in the nanowire after doping were significantly shifted, indicating the change in the original tungsten oxide nanowire chemical state due to the doping. In the photodegradation experiment of methylene blue, the photocatalytic efficiency of manganese-doped and potassium-doped tungsten oxide nanowires was better than that of undoped tungsten oxide nanowires; after 150 min, the degradation efficiency of manganese-doped tungsten oxide nanowires was 17.5% higher than that of undoped tungsten oxide nanowires. In the gas sensing experiment, the single hexagonal phase brought the best gas sensing sensitivity; at 250 °C, the potassium-doped tungsten oxide nanowires improved the response to 20 ppm of ethanol by ~30% as compared with the undoped tungsten oxide nanowires. The synthesized manganese-doped and potassium-doped tungsten oxide nanowires are excellent choices for the photocatalysis of wastewater treatment under visible 1light and for gas sensing of ethanol.

## Figures and Tables

**Table 1:** Electrical resistivity measurements of single nanowires.

	WO_3_ NW	Mn-Doped WO_3_ NW	K-Doped WO_3_ NW
Resistivity	8.27 × 10−6 Ω·m	1.81 × 10−5 Ω·m	1.93 × 10−5 Ω·m

## Supplementary Materials

The following are available online at https://www.mdpi.com/article/10.3390/nano12071208/s1, Figure S1—EDS analysis of (a) Mn-doped WO_3_ NW and (b) K-doped WO_3_ NW. Figure S2—EDS mapping of (a) Mn-doped WO_3_ NW and (b) K-doped WO_3_ NW. Figure S3—Schematic illustration for the preparation of electrical measurement micro-device: (a) Paste copper mesh on a silicon substrate with silicon dioxide; (b) deposit silver as a conductive layer; (c) drip nanowire solution; (d) connect the nanowire with electrodes by FIB. Figure S4—Schematic illustration of electrical resistivity measurements of a single nanowire. (a) The relative position and label of the electrode and nanowire; (b) the electrode under low magnification; (c) SEM image of the nanowire connected to electrodes; (d) HRSEM image of the nanowire. Figure S5—I–V measurements of single WO_3_ nanowire: (a) R13+, (b) R13−, (c) R14+, (d) R14−, (e) R23+, (f) R23−, (g) R24+, and (h) R24−. Figure S6—I–V measurements of single Mn-doped WO_3_ nanowire: (a) R13+, (b) R13−, (c) R14+, (d) R14−, (e) R23+, (f) R23−, (g) R24+, and (h) R24−. Figure S7—I–V measurements of single K-doped WO_3_ nanowire: (a) R13+, (b) R13−, (c) R14+, (d) R14−, (e) R23+, (f) R23−, (g) R24+, and (h) R24. Figure S8—XPS analysis for the tungsten oxide nanowires in W4f energy level: (a) undoped nanowires, (b) Mn-doped nanowires, and (c) K-doped nanowires.
