# Supplementary material for: Chemical Vapor Deposition-Fabricated Manganese-Doped and Potassium-Doped Hexagonal Tungsten Trioxide Nanowires with Enhanced Gas Sensing and Photocatalytic Properties
Source: Nanomaterials (Basel). 2022 Apr 4;12(7):1208. doi: 10.3390/nano12071208 (PMC9002994; doi:10.3390/nano12071208)
Supplement: Supplementary file 1 [file nanomaterials-12-01208-s001.zip › nanomaterials-1628912-supplementary.pdf]

nanomaterials

Supporting Information

Chemical Vapor Deposition-Fabricated Manganese-Doped and Potassium-Doped Hexagonal Tungsten Trioxide Nanowires with Enhanced Gas Sensing and Photocatalytic Properties

Pin-Ru Chen [1], Hsuan-Wei Fu [1], Shu-Meng Yang [1] and Kuo-Chang Lu [1,2]*

[1] Department of Materials Science and Engineering, National Cheng Kung University, Tainan 701, Taiwan; kitty110384@gmail.com (P.-R.C.); n56091556@gs.ncku.edu.tw (H.-W. F.); n56074287@gs.ncku.edu.tw (S.-M.Y.)
[2] Core Facility Center, National Cheng Kung University, Tainan 701, Taiwan
* Correspondence: gkclu@mail.ncku.edu.tw; Tel.: +886-6-275-7575#62920

List of contents

Figure S1｜EDS analysis of (a) Mn-doped WO_3 NW (b) K-doped WO_3 NW.
Figure S2｜EDS mapping of (a) Mn-doped WO_3 NW (b) K-doped WO_3 NW.
Figure S3｜Schematic illustration for the preparation of electrical measurement micro-device (a) paste copper mesh on a silicon substrate with silicon dioxide (b) deposit silver as a conductive layer (c) drip nanowire solution (d) connect the nanowire with electrodes by FIB.
Figure S4｜Schematic illustration of electrical resistivity measurements of a single nanowire (a) The relative position and label of the electrode and nanowire (b) The electrode under low magnification (c) SEM image of the nanowire connected to electrodes (d) HRSEM image of the nanowire.
Figure S5｜I-V measurements of single WO_3 nanowire (a) R_{13+} (b) R_{13-} (c) R_{14+} (d) R_{14-}(e) R_{23+} (f) R_{23-} (g) R_{24+} (h) R_{24-}.
Figure S6｜I-V measurements of single Mn-doped WO_3 nanowire (a) R_{13+} (b) R_{13-} (c) R_{14+} (d) R_{14-}(e) R_{23+} (f) R_{23-} (g) R_{24+} (h) R_{24-}.
Figure S7｜I-V measurements of single K-doped WO_3 nanowire (a) R_{13+} (b) R_{13-} (c) R_{14+} (d) R_{14-}(e) R_{23+} (f) R_{23-} (g) R_{24+} (h) R_{24}.
Figure S8｜XPS analysis for the tungsten oxide nanowires in W4f energy level (a) undoped nanowires (b) Mn-doped nanowires (c) K-doped nanowires.

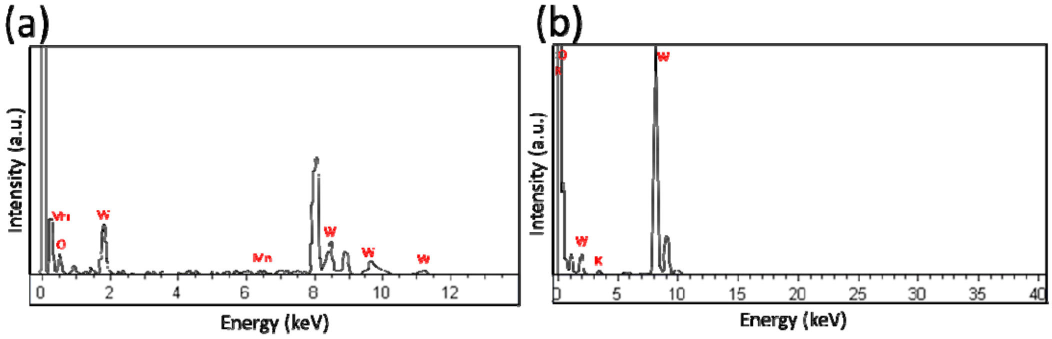

Element	Weight%	Atomic%
O K	6.32	43.39
W L	93.25	55.74
Mn K	0.43	0.87
Totals	100.00	

Element	Weight%	Atomic%
O K	18.81	68.58
K K	4.81	7.18
W L	76.38	24.23
Totals	100.00	

Figure S1. EDS analysis of (**a**) Mn-doped WO_3 NW (**b**) K-doped WO_3 NW.

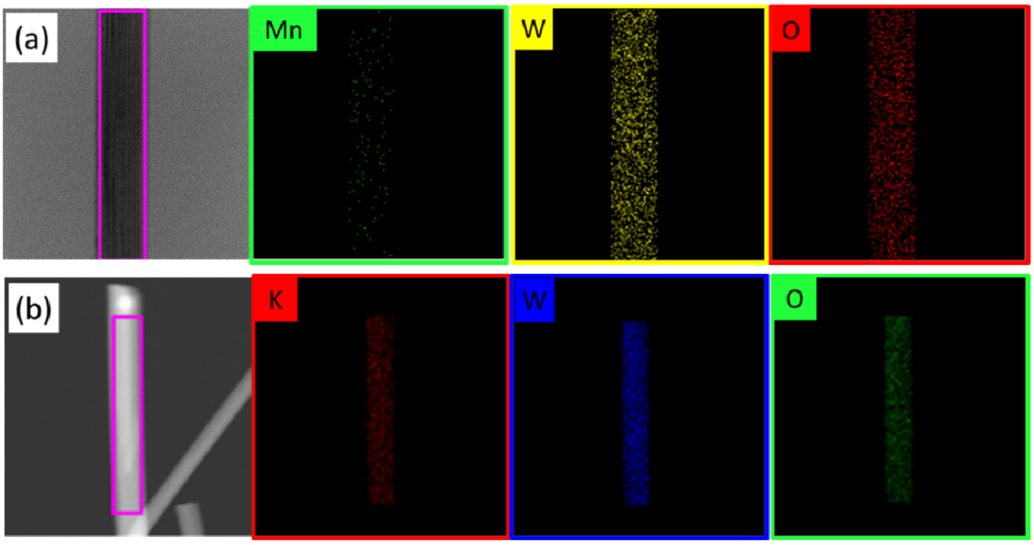

Figure S2. EDS mapping of (**a**) Mn-doped WO_3 NW (**b**) K-doped WO_3 NW.

Figure S3 and S4 are for electrical measurements. The silicon substrate was deposited with silicon dioxide as an insulating layer by an electron beam evaporator, a copper mesh without carbon coating was pasted as a mask, and 200 nm-thick silver was deposited as a conductive electrode. The nanowire was dripped in the center of the copper mesh and connected to four electrodes with FIB.

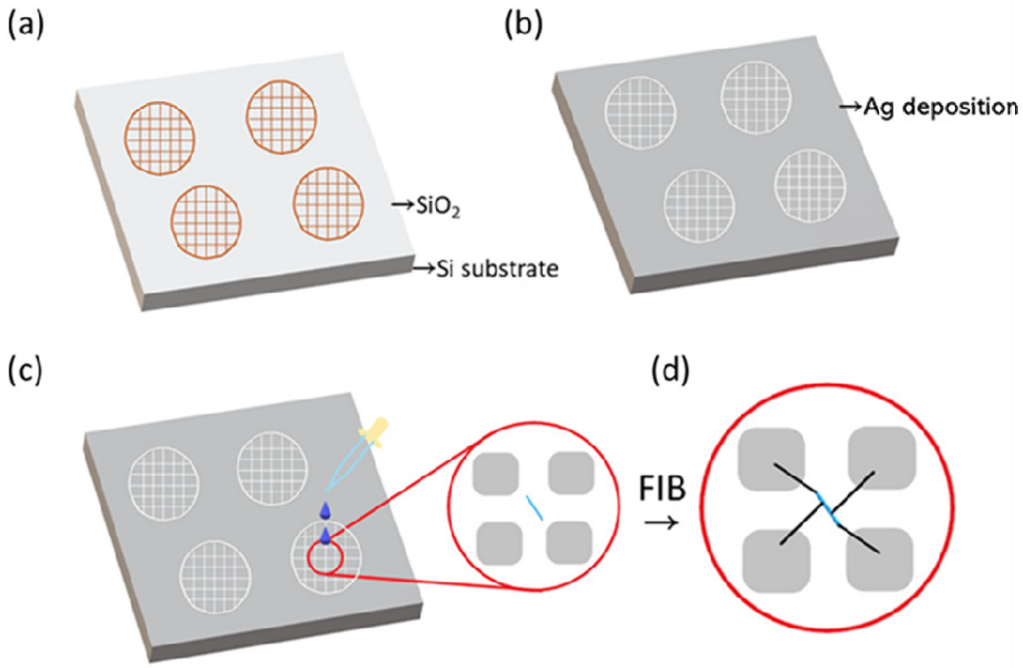

Figure S3. Schematic illustration for the preparation of electrical measurement micro-device (**a**) paste copper mesh on a silicon substrate with silicon dioxide (**b**) deposit silver as a conductive layer (**c**) drip nanowire solution (**d**) connect the nanowire with electrodes by FIB.

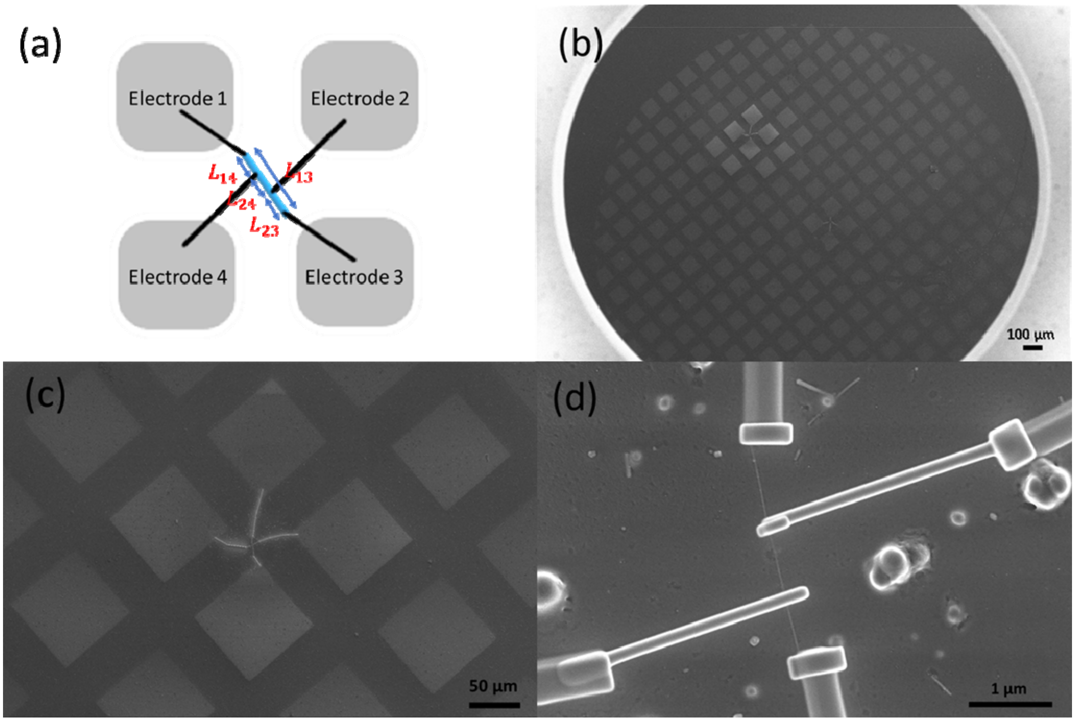

Figure S4. Schematic illustration of electrical resistivity measurements of a single nanowire (**a**) the relative position and label of the electrode and nanowire (**b**) the electrode under low magnification (**c**) SEM image of the nanowire connected to electrodes (**d**) HRSEM image of the nanowire.

We used the single nanowire measurement method reported by Gu et al. [1], which can eliminate the errors from Ohmic and Schottky contacts. Electrical measurements are shown in Figure S6-8.

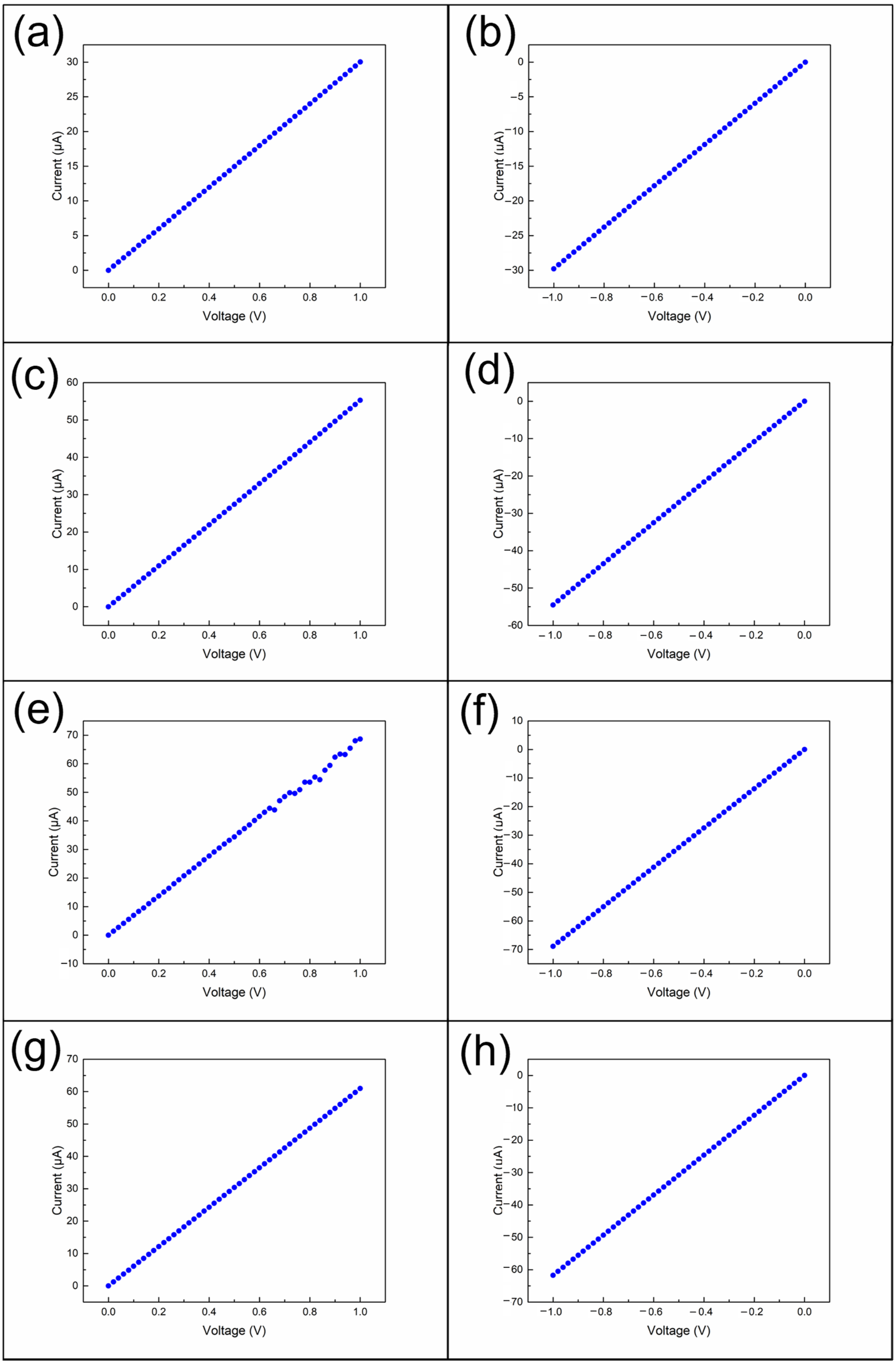

Figure S5. I-V measurements of single WO_3 nanowire (**a**) R_{13+} (**b**) R_{13-} (**c**) R_{14+} (**d**) R_{14-} (**e**) R_{23+} (**f**) R_{23-} (**g**) R_{24+} (**h**) R_{24-}.

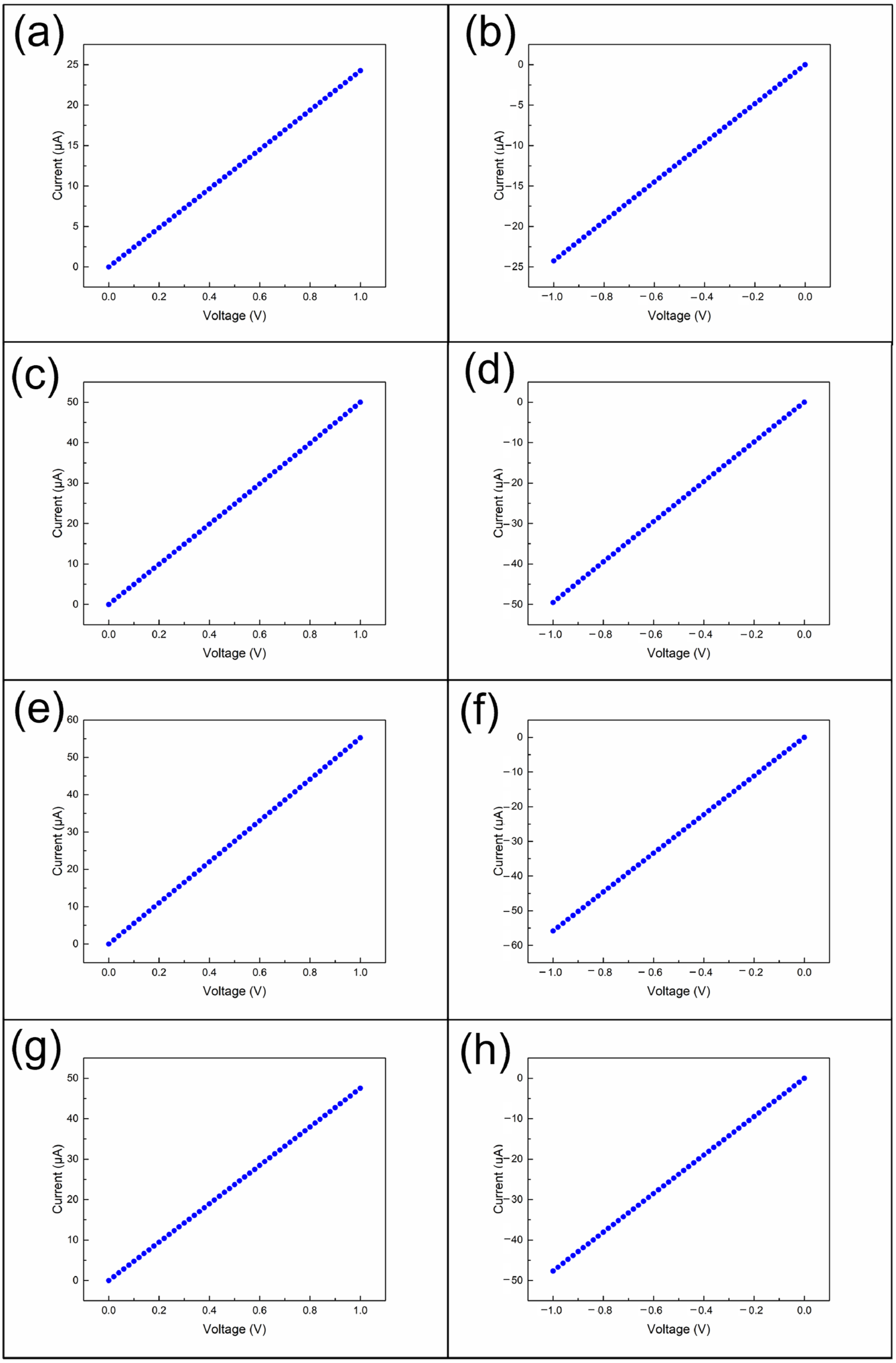

Figure S6. I-V measurements of single Mn-doped WO_3 nanowire (a) R_{13+} (b) R_{13-} (c) R_{14+} (d) R_{14-} (e) R_{23+} (f) R_{23-} (g) R_{24+} (h) R_{24-}.

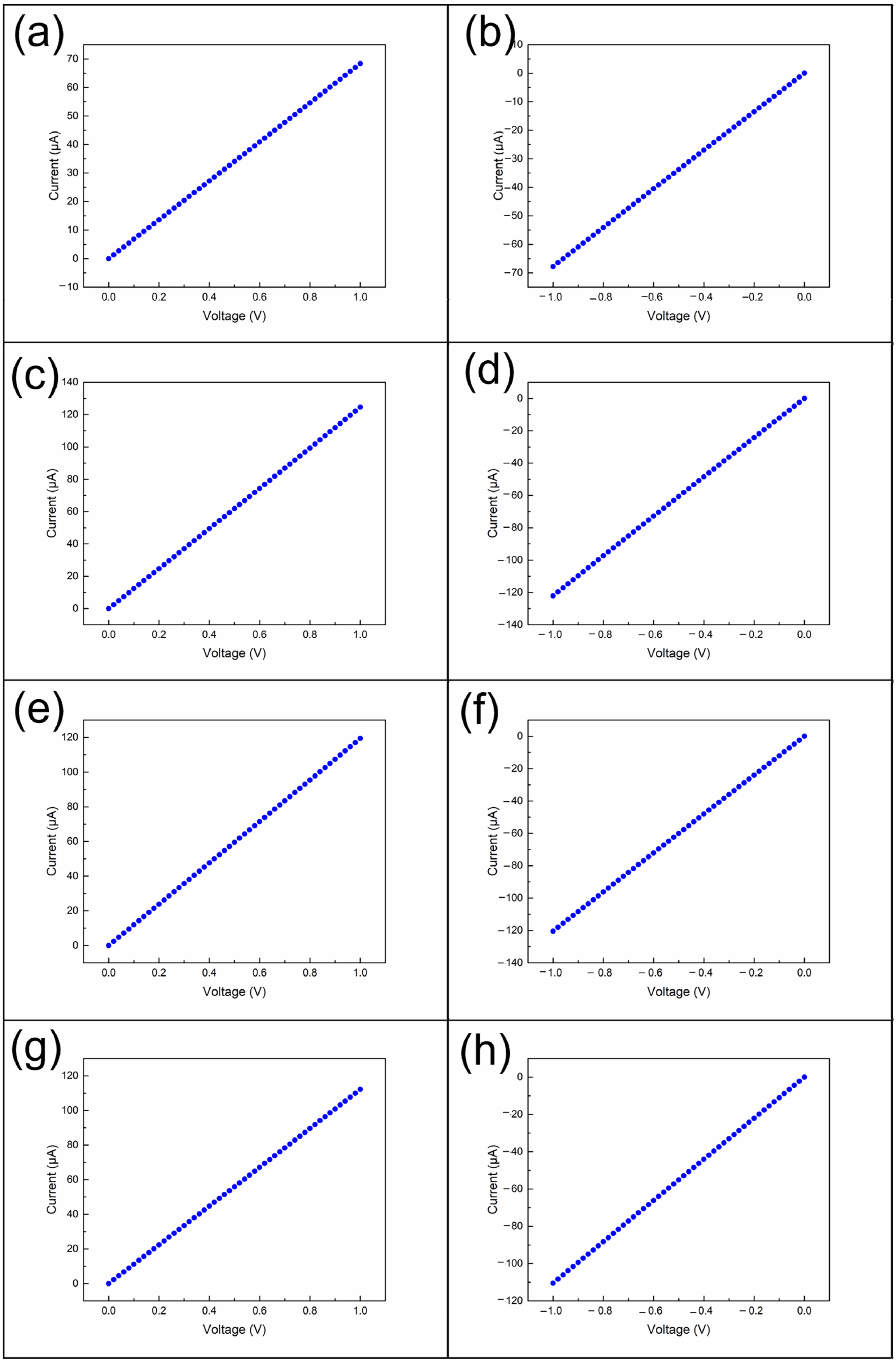

Figure S7. I-V measurements of single K-doped WO_3 nanowire (**a**) R_{13+} (**b**) R_{13-} (**c**) R_{14+} (**d**) R_{14-} (**e**) R_{23+} (**f**) R_{23-} (**g**) R_{24+} (**h**) R_{24-}.

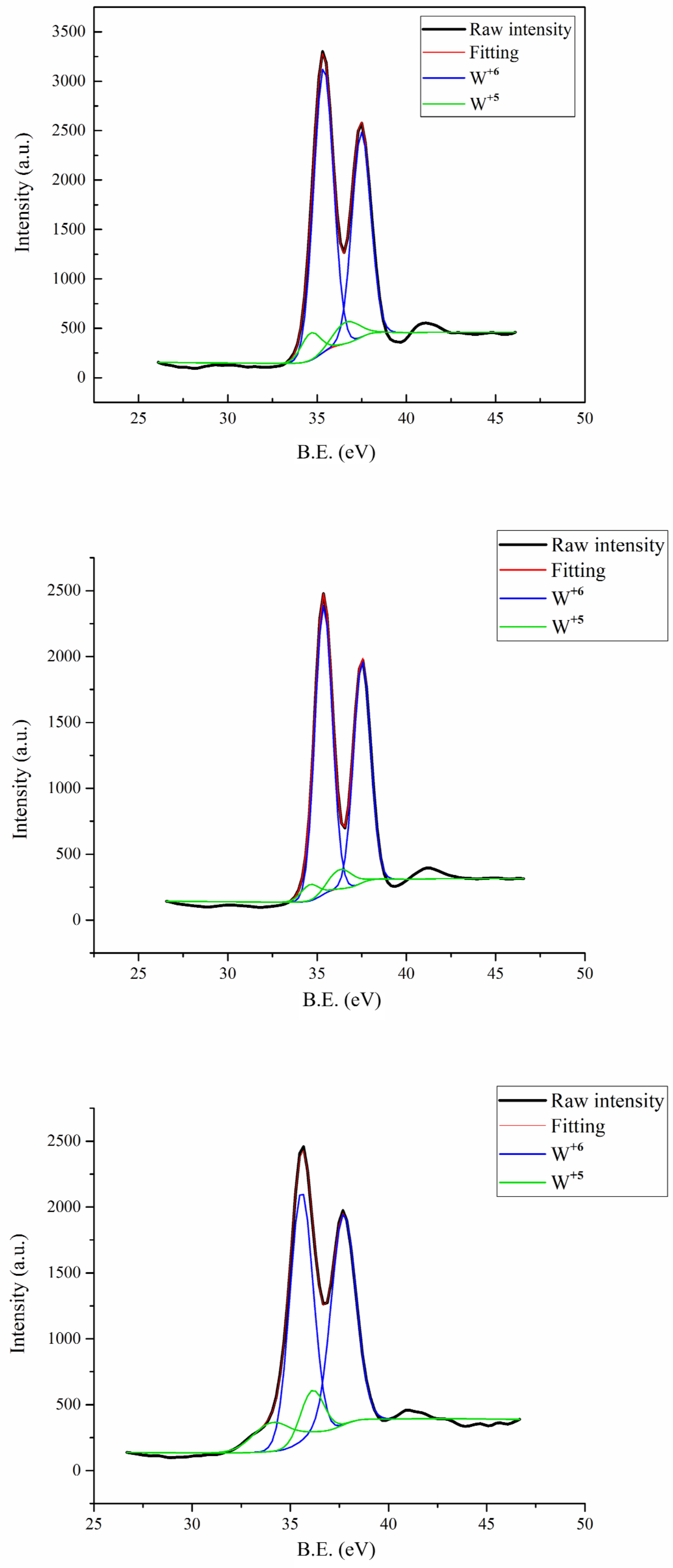

Figure S8. XPS analysis for the tungsten oxide nanowires in W4f energy level (**a**) undoped nanowires (**b**) Mn-doped nanowires (**c**) K-doped nanowires.

Reference

1. Gu, W.; Choi, H.; Kim, K.K. Universal approach to accurate resistivity measurement for a single nanowire: Theory and application. *Appl. Phys.* **2006**, *89*, 253102.
